# Passive heating and glycaemic control in non-diabetic and diabetic individuals: A systematic review and meta-analysis

**DOI:** 10.1371/journal.pone.0214223

**Published:** 2019-03-22

**Authors:** Matthew J. Maley, Andrew P. Hunt, Ian B. Stewart, Steve H. Faulkner, Geoffrey M. Minett

**Affiliations:** 1 Institute of Health and Biomedical Innovation, School of Exercise and Nutrition Sciences, Queensland University of Technology, Brisbane, Australia; 2 Department of Sport and Exercise Science, University of Portsmouth, Portsmouth, United Kingdom; 3 Department of Engineering, School of Science and Technology, Nottingham Trent University, Nottingham, United Kingdom; University of Nortwestern Rio Grande do Sul State (UNIJUI), BRAZIL

## Abstract

**Objective:**

Passive heating (PH) has begun to gain research attention as an alternative therapy for cardio-metabolic diseases. Whether PH improves glycaemic control in diabetic and non-diabetic individuals is unknown. This study aims to review and conduct a meta-analysis of published literature relating to PH and glycaemic control.

**Methods:**

Electronic data sources, PubMed, Embase and Web of Science from inception to July 2018 were searched for randomised controlled trials (RCT) studying the effect of PH on glycaemic control in diabetic or non-diabetic individuals. To measure the treatment effect, standardised mean differences (SMD) with 95% confidence intervals (CI) were calculated.

**Results:**

Fourteen articles were included in the meta-analysis. Following a glucose load, glucose concentration was greater during PH in non-diabetic (SMD 0.75, 95% CI 1.02 to 0.48, P < 0.001) and diabetic individuals (SMD 0.27, 95% CI 0.52 to 0.02, P = 0.030). In non-diabetic individuals, glycaemic control did not differ between PH and control only (SMD 0.11, 95% CI 0.44 to -0.22, P > 0.050) and a glucose challenge given within 24 hours post-heating (SMD 0.30, 95% CI 0.62 to -0.02, P > 0.050).

**Conclusion:**

PH preceded by a glucose load results in acute glucose intolerance in non-diabetic and diabetic individuals. However, heating a non-diabetic individual without a glucose load appears not to affect glycaemic control. Likewise, a glucose challenge given within 24 hours of a single-bout of heating does not affect glucose tolerance in non-diabetic individuals. Despite the promise PH may hold, no short-term benefit to glucose tolerance is observed in non-diabetic individuals. More research is needed to elucidate whether this alternative therapy benefits diabetic individuals.

## Introduction

Frequent passive heating (PH), often referred to as Waon therapy, hot-tub therapy or thermal therapy, may provide health benefits for those who are in diseased and non-diseased states [1–6]. Chronic use of Finnish saunas (80 °C– 100 °C air, < 20% relative humidity) is associated with a reduced risk of dementia, stroke, respiratory disease, hypertension, fatal cardiovascular and all-cause mortality events [7–11]. The mechanisms responsible for how PH maintains health in those free of disease and improves health in diseased conditions are not completely clear. Current evidence suggests PH elicits improvements in shear patterns (increase in antegrade shear), microvascular function (endothelial-dependent), lipid profiles, reduced arterial stiffness and blood pressure, as well as reduced heart rate and deep body temperature during heat stress [12–21]; responses that are also evident, albeit to a greater extent, following regular physical activity.

The therapeutic effects of PH on those experiencing poor glycaemic control has not been thoroughly investigated in humans. Globally, it was estimated 422 million adults aged over 18 years were living with diabetes in 2014 [22], equating to ~8.5% of the world’s population. Type 2 diabetes mellitus (T2DM), accounting for ~90% of individuals with diabetes, is associated with high blood glucose, insulin resistance and increased insulin secretion [23,24]. Glycogen stored in the liver can be released into the bloodstream as blood sugar (glycogenolysis), with cells able to metabolise the glucose or store for later needs. This process maintains blood sugar in between meals but is not as tightly regulated in those with diabetes and poor glycaemic control. In these compromised conditions, blood glucose rises due to the insulin insensitivity, resulting in more insulin being released. T2DM is a progressive disease, with prolonged untreated states leading to pancreatic beta-cell damage and loss of insulin secretion [24]. Lifestyle interventions (e.g. diet assessment, promotion of physical activity) may attenuate or even reverse the complications associated with T2DM [25,26]. Despite the benefits associated with these interventions, adherence is often poor and, in some cases, not possible. Alongside lifestyle interventions, drug therapy is often prescribed but may carry unwanted side effects [27].

Non-pharmaceutical interventions, such as PH, may benefit people with diabetes and those with poor glycaemic control. Supporting this viewpoint, Hooper [6] invited eight participants with T2DM to sit in warm water (38 °C– 41 °C) for 30 minutes a day, six days a week over three weeks. At the end of the three weeks, fasting glucose was reduced, but more importantly, haemoglobin A1c (HbA_1c_) was reduced by 1%. Changes in HbA_1C_ of the 1% magnitude reported are clinically important as they are associated with a 21% reduction in all-cause diabetes-related deaths [28]. Notably, however, no control group was included in this study and differences in age, sex and disease severity amongst participants potentially confound the results.

Resting in warm environments may induce hormonal changes that may influence glycaemic control [4,29]. While insulin concentrations may not change during PH, thyroid hormone, growth hormone, noradrenaline and adrenaline concentrations may rise to elicit greater concentrations of blood glucose [30–32]. Acutely raising blood glucose concentrations is not of benefit but PH may elicit other changes to reduce the blood glucose concentration. Muscle temperature and blood flow may rise if heating is sufficient, which may acutely promote muscle glucose uptake [33,34]. The mechanisms responsible for the chronic reduction in fasting glucose and HbA_1C_ may be multi-factorial, but heat shock proteins (HSP) may play a pivotal role [35]. Upon physiological stress (e.g. heat, hypoxia, cancer) there is an increase in the amount of unfolded and misfolded proteins, causing cell damage [36–38]. The physiological stress is accompanied by a heat shock response, which triggers the release of HSP. The increased number of HSP available help facilitate correct folding of proteins, thus preventing cell damage. Both human and murine diabetic models are characterised by low intracellular (i) and high extracellular (e) HSP levels [39–45], promoting a pro-inflammatory state that reduces insulin sensitivity [46,47]. iHSP has direct protective effects whereas high eHSP is linked with insulin resistance [48,49]. Restoration of iHSP levels in diabetic models and subsequent favourable glycaemic control are mediated, in part, through reductions in inflammatory cytokines, c-Jun N-terminal kinase and IkappaB kinase, both associated with the inhibition of insulin signalling [50,51]. Importantly, PH has been shown to increase iHSP levels in diabetic and obese human and murine models [39,52,53].

Considering these findings, it would seem prudent to study PH and its effect on glycaemic control further. Indeed, reviews have highlighted how PH may benefit individuals with diabetes or those who are insulin resistant [27,35,45,46,54–58], but to date, there is no systematic search, review and meta-analysis of PH and glycaemic control in diabetic and non-diabetic individuals. Therefore, the purpose of this study was to review and conduct a meta-analysis of published literature relating to PH and glycaemic control.

## Methods

### Search strategy

**Fig 1:**
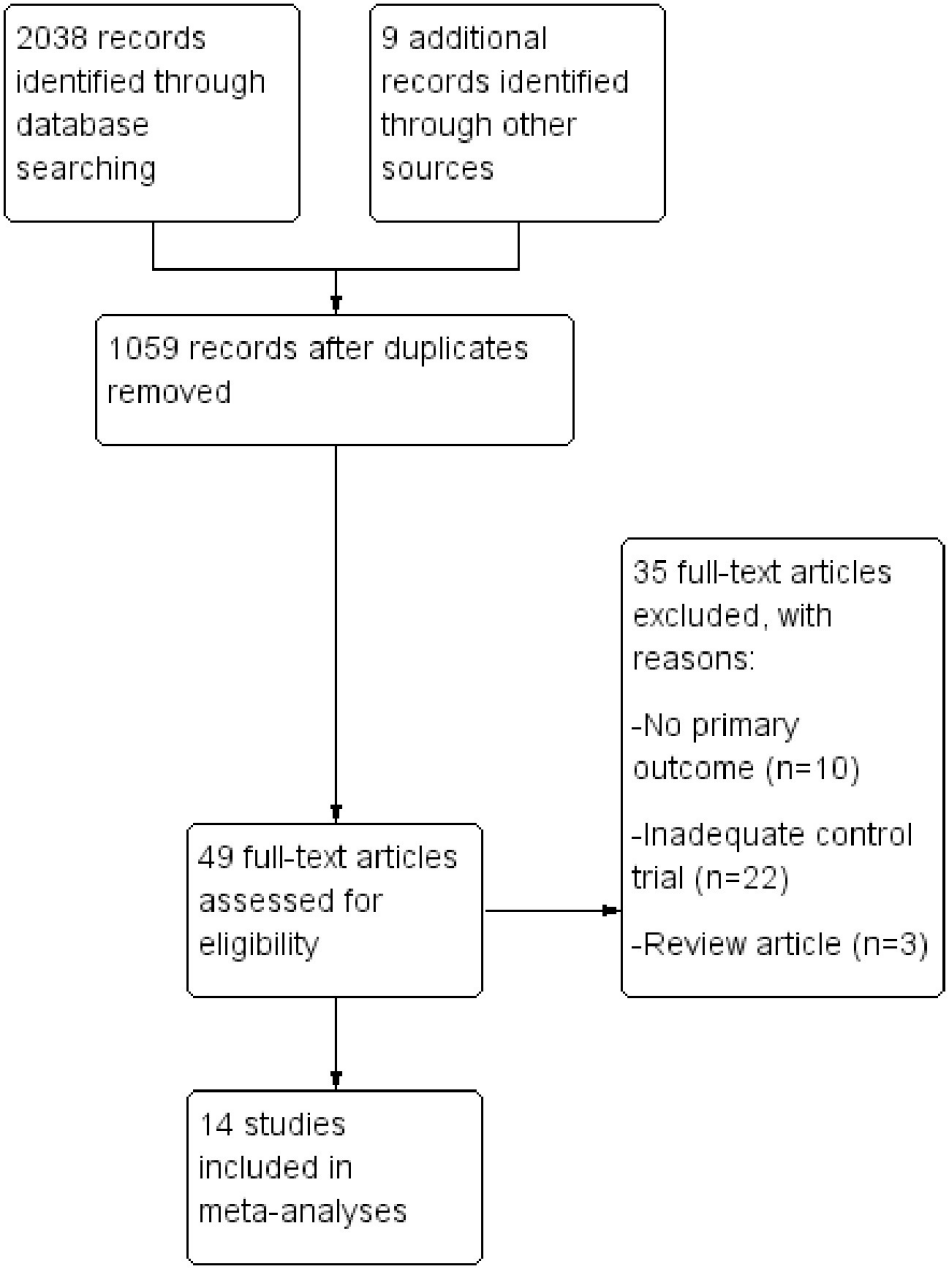
PRISMA flowchart.

An electronic literature search was conducted in July 2018 using PubMed, Embase, and Web of Science. Searches were performed using Boolean operators and the following key terms and their combinations: *glucose*, *insulin*, *diabetes*, *passive heating*, *sauna*, *thermal therapy*, *warm water*, *warm air*, *thermotherapy*, and *hot water immersion*. The reference lists of all included studies were also examined to identify potentially relevant data sets that were not found in the original search (Fig 1).

### Study inclusion and exclusion criteria

Studies were included in the review where: 1) a PH intervention, defined as any technique designed to increase body temperature using non-exercise models, was applied; 2) the experimental design included a non-heating control trial; 3) at least one primary outcome measure (i.e. glucose or insulin) was reported; 4) participants were human adults (i.e. aged ≥18 years), and 5) data were published in a peer-reviewed journal. There were no restrictions applied to the PH mode (e.g. water or air), exposure duration, participant sex, health status or study setting. Studies involving exercise in combination with PH were excluded.

### Selection criteria

Titles and abstracts returned by the search strategy were screened independently by two authors (MM, GM) to remove those that were outside of the scope of the review. Full-text of papers that potentially met the review inclusion criteria were obtained. Disagreements between authors regarding study inclusion were resolved by consensus or a third party (AH).

### Data extraction

A customised form was used to extract relevant data on methodological design independently, eligibility criteria, interventions, participant descriptors, comparisons and outcome measures by two authors (MM, AH). The outcome measures extracted included 1) blood glucose and insulin; and 2) complications or adverse effects experienced attributable to the intervention. The authors of original investigations were contacted via email, as required, to clarify any queries relating to data or study characteristics as required. Any disagreement between the review authors extracting data was resolved by consensus or a third party (GM).

### Risk of bias assessment

**Fig 2:**
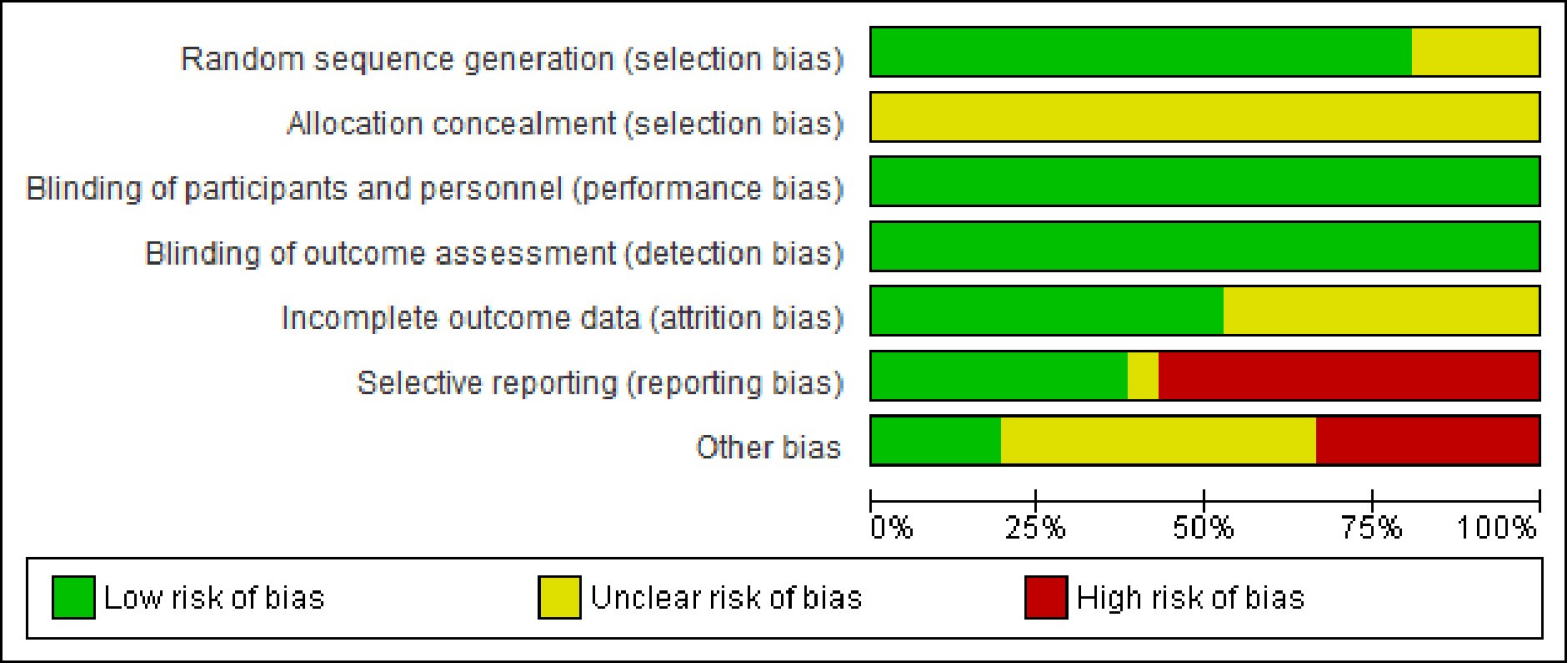
Risk of bias summary.

Risk of bias assessment was independently conducted in accordance with the Cochrane Handbook for Systematic Reviews of Interventions [59] by two authors (MM, AH). Potential sources of bias were classified as high, low or unclear in the areas of sequence generation, allocation concealment, blinding of participants and personnel, blinding of outcome assessment, incomplete outcome data, selective outcome reporting, and other bias. These outcomes were visually summarised (Fig 2), with any disagreement between the authors’ interpretation resolved by consensus or a third party (GM).

### Statistical analyses

To measure the treatment effect, standardised mean differences (SMD) with 95% confidence intervals (CI) were calculated and analysed using a random effects model. Missing data prompted an email request to the study authors seeking this data and/or clarification as to why data were missing. The absence of standard deviations that could not be sourced from authors were calculated from available statistics as per Higgins and Green [59]. Further, where necessary, data were manually extracted from figures using WebPlotDigitizer [60]. Heterogeneity between comparable trials was evaluated using the I^2^ statistic. Values of I^2^ were interpreted using the following scale [59]: 0% to 40%, might not be important; 30% to 60%, moderate heterogeneity; 50% to 90%, substantial heterogeneity; and 75% to 100%, considerable heterogeneity.

## Results

The search strategy identified a total of 2038 records (Fig 1). We also found nine potentially eligible studies from additional reference searches in these papers. Following removal of duplicates (n = 988), 1059 titles and abstracts were screened which resulted in 49 full-text articles being retrieved for eligibility. Articles were included in full-text screening if the abstract discussed PH in humans relating to either non-diabetic or diabetic individuals. After full-text screening, another 35 articles were excluded, mostly due to no relevant primary outcome or inappropriate control trial. Consequently, 14 articles were included in the final meta-analysis.

### Study characteristics

Table 1 shows the characteristics of the included studies. The sample size varied from six [30,61,62] to 32 [63]. In two studies, both non-diabetic and diabetic individuals were examined [63,64], while nine studies focused on non-diabetic individuals [30,62,65–71] and three studies focused on diabetic individuals [61,72,73]. The studies using diabetic participants described their cohort as non-insulin-dependent [63], insulin-dependent [61,72], type 1 [73] and T2DM [64]. The participants described as non-insulin-dependent received treatment with small doses of oral sulfonylureas [63]. The studies utilising insulin-dependent diabetics were described as receiving between 20 U to 27 U of intermediate or intermediate plus rapid-acting insulin in one of two injections per day [61,72], or 26 U to 56 U of long or long plus rapid-acting insulin in one or two injections per day [73]. The T2DM participants were taking Metformin [64].

In four studies, both male and female individuals were examined [63,64,67,71], while ten studies examined males only [30,61,62,65,66,68–70,72,73]. The mean age of the participants varied between 19 years [73] and 56 years [63].

**Table 1:** Characteristics of included studies.

First author, year	Population^a^	Fasted?	Glucose load?	Insulin given?	Time between glucose/insulin and heating (minute)	Control protocol	Passive heating protocol
Akanji 1987	22 non-diabetic • 6l males/4 females, normal weight, 47(13) years of age • 6 males/6 females, overweight, 43(13) years of age	Yes	960 kcal meal	No	0	23 °C air, 120 minutes	33 °C air, 120 minutes
	5 males/1 female non-diabetic, normal weight, 19–57 years of age	Yes	75 g of glucose	No	0	23 °C air, 120 minutes	33 °C air, 120 minutes
Akanji 1991	16 non-diabetic • 4 male/4 female, normal weight, 45(6) years of age • 4 male/4 female, overweight, 42(6) years of age 16 diabetic • 4 male/4 female, normal weight, 54(11) years of age • 4 male/4 female, overweight, 56(11) years of age	Yes	75 g of glucose	No	0	23 °C air, 120 minutes	33 °C air, 120 minutes
Dumke 2015	11 males non-diabetic, normal weight, 22(3) years of age	Yes	Glucose given at 1.8 g·kg^–1^ of body mass	No	0	22 °C air, 180 minutes	43 °C air, 180 minutes
Faure 2016	Study A: 10 males, non-diabetic, normal weight, 21(2) years of age	Yes	62 kcal meal	No	Given 30 minutes post-heating	22 °C for 40 minutes	31 °C for 40 minutes
	Study B: 12 males, non-diabetic, normal weight, 20(2) years of age	Yes	75 g of glucose	No	0	22 °C air, 180 minutes	31 °C air, 180 minutes
Frayn 1989	4 males/2 females, non-diabetic, normal weight, 20–40 years of age	Yes	75 g of glucose	No	0	23 °C air, 120 minutes	33 °C air, 120 minutes
Jezova 1998	9 males, non-diabetic, normal weight, 23–25 years of age	Yes	No	No	NA	22–24 °C air, 45 minutes	53 °C sauna, 45 minutes
Jurcovicova 1980	Experiment 1: 6 males, non-diabetic, normal weight, 23–27 years of age	Yes	Glucose given at 1 g·kg^–1^ of body mass	No	Given immediately following heating	30 °C water to neck, 30 minutes	40 °C water to neck, 30 minutes
	Experiment 2: 6 males, non-diabetic, normal weight, 23–27 years of age	Yes	100 g of glucose	No	Given 90 minutes post-heating	30 °C water to neck, 30 minutes	40 °C water to neck, 30 minutes
Koivisto 1980	8 males, diabetic, normal weight, 34(11) years of age	Yes	430 kcal meal	10 U Actrapid & 6–40 U Monotard given immediately before meal.	60 minutes	22 °C air, 60 minutes	85 °C sauna, 60 minutes
Koivisto 1981	6 males, diabetic, normal weight, 29(7) years of age	Yes	280 kcal meal	6 U Actrapid given immediately before meal.	0	20 °C air, 240 minutes	35 °C air, 240 minutes
Koivisto 1983	8 males, diabetic, normal weight, 19(8) years of age	Yes	430 kcal meal	14 U Semilente given immediately before meal.	60 minutes	22 °C air, 60 minutes total	85 °C sauna, 60 minutes total
Linnane 2004	7 males, non-diabetic, normal weight, 27(8) years of age	No	No	No	NA	Lay down in empty bath for 15 minutes then sat in 20 °C air for 30 minutes	43 °C water to neck for ~16 minutes, then sat in 44 °C air for ~30 minutes
Moses 1997	7 males, non-diabetic, normal weight, 24(4) years of age	Yes	75 g of glucose	No	0	25 °C air, 120 minutes	35 °C air, 120 minutes
Rivas 2016	• 2 male/7 female non-diabetic, overweight, 41(14) years of age 3 male/6 female diabetic, overweight, 50(12) years of age	Yes	75 g of glucose	No	Given 24-hours post-heating	24 °C air, 120 minutes	39 °C water, 120 minutes
Tatar 1985	• 6 males, non-diabetic, normal weight, 22–24 years of age	Yes	100 g of glucose	No	15 minutes	23 °C air, 30 minutes	85 °C sauna for 30 minutes

^a^Age given in mean (SD); for missing mean (SD), the range is specified. Participants distributed by sex if data were available. Normal weight defined as a body mass index ≤ 25.

Regarding methodology, all participants were fasted, except one study [68]. A glucose load (meal or glucose solution) was given before PH and control trials in seven studies using non-diabetic individuals [62,63,65–67,69,71]. A glucose load and insulin were given before PH and control trials in three studies using diabetic individuals [61,72,73]. No glucose or insulin was given before PH and control in four experiments [30,66,68,70]. Three experiments included data where a glucose load was given immediately [30], 120 minutes [30] or 24-hours post-heating [64]. Eleven studies used air (31 °C to 85 °C) to heat participants [61–63,65–67,69–73], while two studies used water (39 °C– 40 °C) [30,64], and the other used a mix of water (43 °C) and then air (44 °C) [68]. Heating duration varied from 30 minutes [30,62] to 240 minutes [61].

All studies measured glucose concentration from venous blood samples. Insulin concentration was measured in five studies using non-diabetic individuals during PH and control trials following a glucose load [62,65–67,69].

### Risk of bias

Risk of bias via the Cochrane Collaboration’s tool indicated that most of the information was from studies with low or unclear bias (Fig 2). Despite not being able to blind participants to a hot environment, performance bias and detection bias was low for all studies as participants were considered unable to change their glucose concentration consciously. Other biases included unclear or inappropriate statistical analyses and unclear handling, storage and analyses of blood samples.

### Meta-analysis outcome

#### Glucose concentration

**Fig 3:**
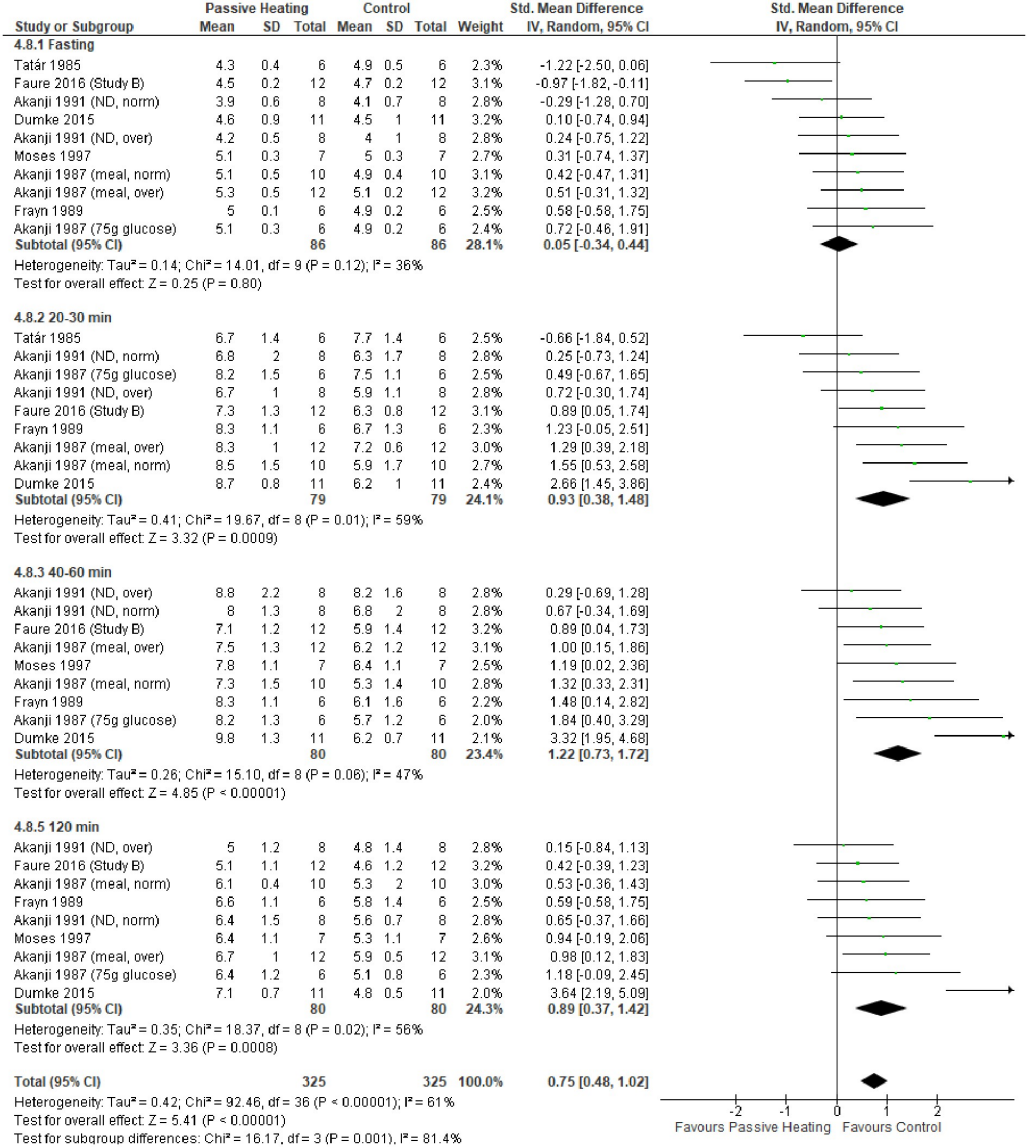
Effects of control and passive heating trials on glucose concentration (mmol/L) in non-diabetic individuals following a glucose load. ND, non-diabetic; D, diabetic; norm, normal weight; over, overweight.

**Fig 4:**
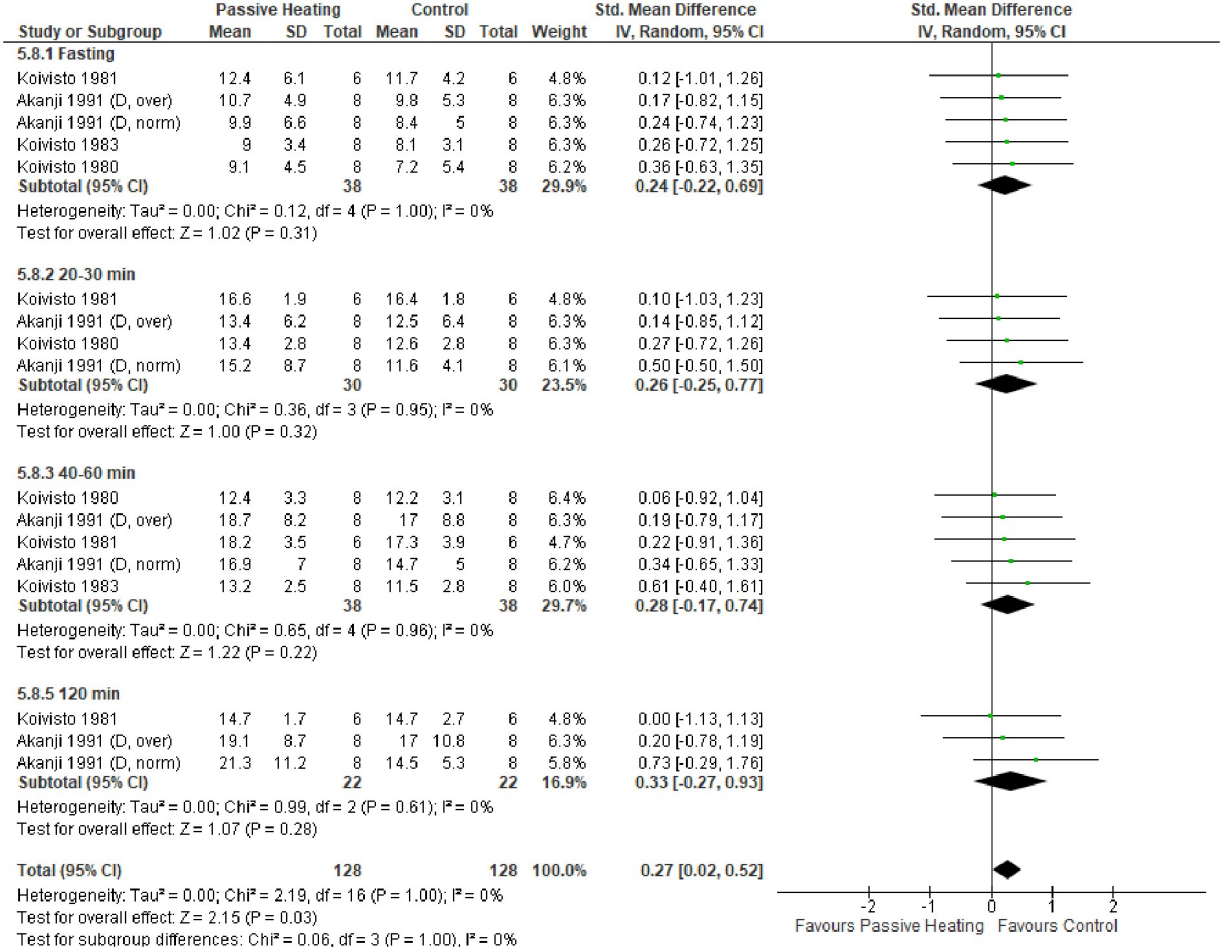
Effects of control and passive heating trials on glucose concentration (mmol/L) in diabetic individuals following a glucose load. ND, non-diabetic; D, diabetic; norm, normal weight; over, overweight.

A summary of individual studies and meta-analysis for glycaemic control following a glucose load are shown in Figs 3 and 4. Fasting glucose concentration did not differ between control and PH in both non-diabetic (Fig 3) and diabetic individuals (Fig 4). Compared with control, glucose concentration was greater after 20–30 minutes of PH in non-diabetic individuals, which was a consistent observation at 40–60 minutes and 120 minutes of PH (Fig 3). In diabetic individuals, glycaemic control did not differ at any time point between PH and control trials, but the pooled overall effect was statistically significant, highlighting the potentially greater glucose concentration during PH (Fig 4).

**Fig 5:**
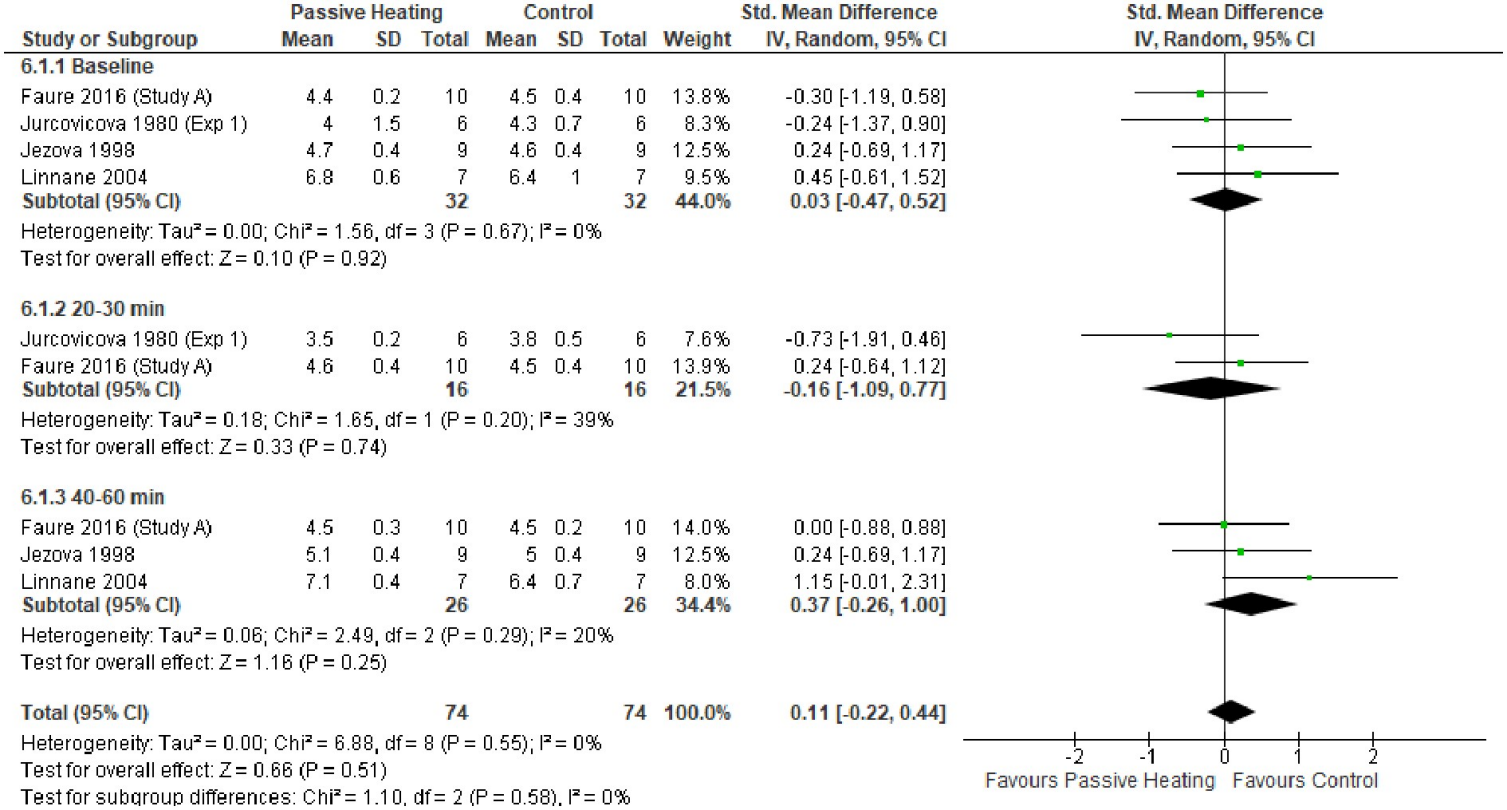
Effects of control and passive heating trials on glucose concentration (mmol/L) in non-diabetic individuals.

**Fig 6:**
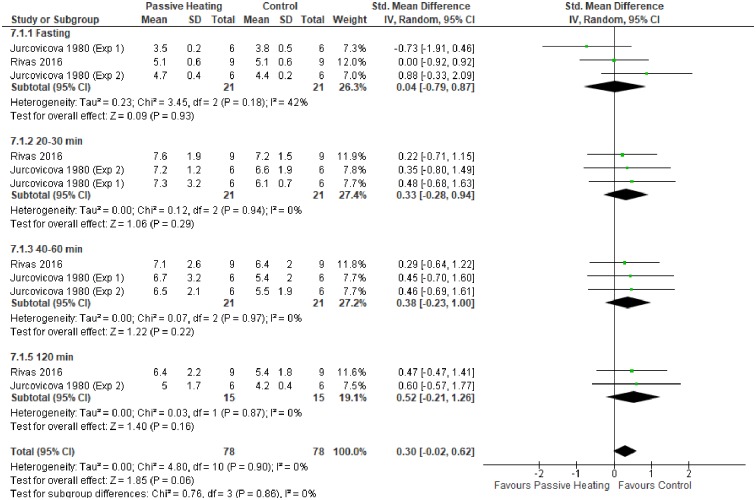
Effects of a glucose load on glucose concentration (mmol/L) after control and passive heating trials in non-diabetic individuals.

When analysing data from experiments that did not administer a glucose load, glycaemic control did not differ between PH and control trials (Fig 5). Similarly, glycaemic control did not differ between PH and control trials when a glucose challenge was administered post-heating (Fig 6).

#### Insulin concentration

**Fig 7:**
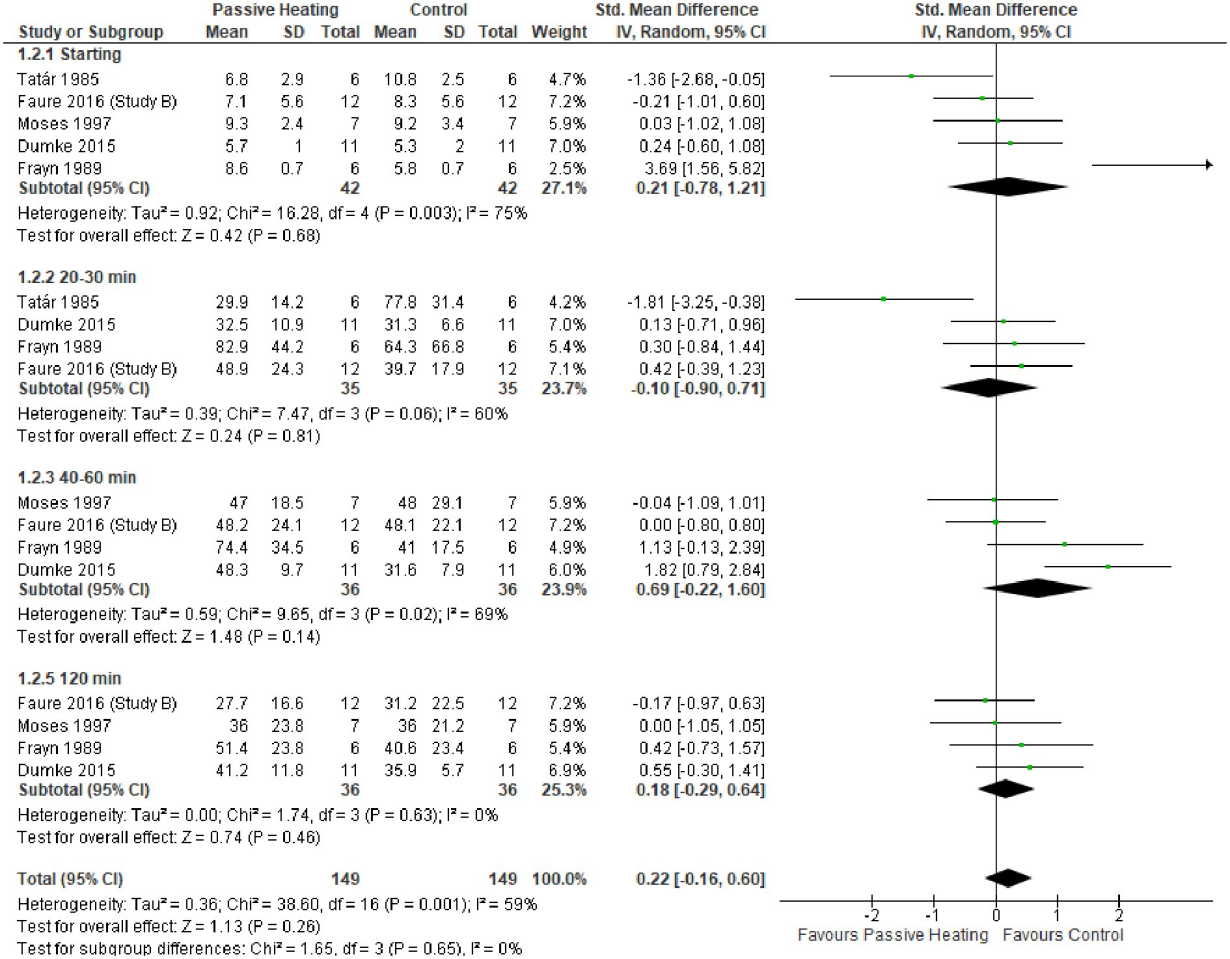
Effects of control and passive heating trials on insulin concentration (μU/mL) in non-diabetic individuals following a glucose load.

A summary of individual studies and meta-analysis of insulin concentration following a glucose challenge is shown in Fig 7. At each time point, insulin concentration was similar between control and PH.

## Discussion

The primary aim of this meta-analysis was to investigate the effect of PH on glycaemic control in diabetic and non-diabetic individuals. Collectively, the meta-analysis showed PH resulted in a greater glucose concentration in diabetic and non-diabetic individuals (Figs 3 and 4). In contrast, glycaemic control does not differ between PH and control trials without a glucose load (Fig 5). Finally, no favourable glycaemic control was observed following a glucose challenge within 24 hours of a single bout of PH (Fig 6).

Hormone changes may, in part, affect blood glucose concentrations during PH [4,29]. Considering glycaemic control does not differ between PH and control trials conducted without a glucose challenge (Fig 5) and insulin concentration does not differ during PH preceded with a glucose load (Fig 7), it is likely other factors may be at play. Arterialisation of venous blood [67] may be another contributing factor. Arterial blood glucose is consistently greater than venous blood glucose, but the difference between a vein in a heated hand and arterial samples is substantially smaller [74] owing to the opening of the arterio-venous anastomoses. This phenomenon presents a problem for oral glucose tolerance tests conducted in varying environments as the sampling technique may be a limiting factor. No study included in this meta-analysis used the heated hand technique [75]. Future studies should be mindful that arterialisation of venous blood may provide a methodological limitation to venous blood glucose sampling when comparing PH and thermoneutral trials. Heating the hand in both thermoneutral and PH trials may circumvent this limitation.

In contrast to non-diabetic individuals, glycaemic control was similar in PH and control trials at individual time points in diabetic individuals (Fig 4). However, the pooled overall effect indicated PH may elicit greater glucose concentrations. It is possible the reduced glucose extraction due to insulin resistance is partly responsible; thus arterialisation of venous blood yields minimal difference. Nevertheless, differences in hormonal responses influencing glucose output and uptake cannot be ruled out.

Providing a glucose challenge post-heating may be a more appropriate research design considering hormonal changes and arterialisation of venous blood may confound outcomes during PH. Glucose tolerance tests are also regularly conducted post-exercise, highlighting an insulin sensitising effect improving glycaemic control that is a relatively short-lived phenomenon (<48 hours) [76]. Limited studies were available for meta-analysis of post-heating glucose tolerance, with PH showing no beneficial effect on glucose tolerance immediate [30], 120 minutes [30] and 24 hours post-heating [64] in non-diabetic individuals. Rivas et al. [64] did also measure glucose tolerance post-heating in T2DM individuals but, again, found PH not to influence glycaemic control. It is possible, as the author’s state [64], they may have missed the window for improved insulin sensitivity or participant’s continued medication may have influenced the glycaemic response. It is clear more work is needed with diabetic individuals to investigate glycaemic control following a single-bout of PH.

Our systematic search of the literature found no chronic randomised control trials investigating the effect of PH on glycaemic control in people with diabetes. Considering Hooper [6] provided evidence of the benefits of PH for those with T2DM nearly 20 years ago, it is surprising to find no other chronic study has been conducted. In the meantime, pharmaceutical agents have been developed to stimulate HSP production, replicating the response observed during PH and exercise [27,35,38,54]. Aside from Hooper [6], only three studies have investigated the effect of chronic PH in individuals with diabetes and other diseased states [15,77]. Specifically, PH (20 minutes, 3 days per week, over 3 months) improved perceived quality of life in people with T2DM [77], while Imamura et al., [15] reported fasting glucose was reduced with PH (45 minutes, 7 days a week, over 2 weeks) in those with coronary risk factors (e.g. obesity, diabetes, hypertension). However, Masuda [21] reported no change in fasting glucose associated with PH (45 minutes, 7 days a week, over 2 weeks) in those with coronary risk factors. The reason for the discrepancy between the latter two studies is not clear.

Favourable glycaemic control following PH was initially attributed to an increased muscle blood flow facilitating glucose uptake [6,15]. Given muscle blood flow may increase during PH [78] and an increase in muscle blood flow may independently facilitate glucose uptake [34], the mechanism is logical. However, the present data show that PH does not acutely benefit glycaemic control in diabetic individuals, thus not supporting the proposed mechanism. Despite this, it is important to highlight the scarcity of data investigating PH and glycaemic control and, in particular, the PH use in diabetic individuals. Based on the limited human data available and current animal models it could be hypothesised that as an individual continues PH sessions over days and weeks, basal iHSP levels rise, eHSP levels fall. Alongside the increased release of nitric oxide and glucose transporter expression, the ratio change between iHSP/eHSP associated with PH will reduce inflammatory cytokines which may improve insulin signalling to aid glycaemic control and reduce the high insulin output [39,45,50–52,79–83]. Other factors such as improved appetite regulation (i.e. increased leptin concentration) and fat mass loss have been shown to occur with PH [6,64] and may work synergistically to improve glucose homeostasis.

If an increase in iHSP is necessary for improvements in glucose homeostasis in diabetic individuals, then a sufficient heating stimulus is required. Exposure to 43 °C– 53 °C air for 60 minutes increased body temperature by ≤0.5 °C [65,70], while immersion in 39 °C water for the same time increased temperature to ~38.5 °C (~1.6 °C difference versus control) [64]. A deep body temperature rise of >0.8 °C has been shown to increase eHSP concentrations [84–86], but it is unknown whether this modest rise in deep body temperature is a great enough stimulus to increase iHSP concentrations. iHSP was increased in murine models where rectal temperature was held at 41.5 °C for ~20 minutes [39,53], and where human whole-blood was incubated for two hours at 42 °C [52]. However, it is not ethically acceptable to maintain rectal temperature >39.5 °C in an attempt to elicit iHSP. Considering the impaired thermoregulatory control in individuals with diabetes [87], finding the lowest thermal stress required for health benefits should be a focus of future work.

No adverse events were noted in the studies reported in this meta-analysis. It is possible, however, with inappropriate PH protocols such an event may occur. For example, PH increases absorption of exogenously delivered insulin which could increase the likelihood of hypoglycaemic events [72,88]. Thermal sensations and thermoregulation may also be impaired in diabetic individuals [87,89] which could lead to burns and heat-related illness.

The strength of the meta-analysis presented here is that it combined data from 14 studies to estimate the effect of PH on glycaemic control with more accuracy than could be achieved in a single study. The risk of bias of the included studies appears low to unclear (Fig 2). If blinding of the participant and outcome assessment are ignored, then the risk of bias is mostly unclear. Importantly, there was a high risk of bias in selective reporting where authors simply failed to report variables or reported only ‘good responders’ [30]. However, the main limitation of this meta-analysis was the methodological differences amongst studies, including participants (e.g. duration of diabetes, age, sex), protocols (e.g. duration and modality of heating) and outcome assessment (e.g. timing of glucose measurement). These differences increase the heterogeneity in outcome measures. To account for heterogeneity, a random-effects model was utilised in the present meta-analysis; with more research in this area future analyses will be more robust and allow subgroup analyses.

In conclusion, this meta-analysis reveals an unclear picture of how PH may benefit glycaemic control in non-diabetic and diabetic humans. In non-diabetic individuals, glucose intolerance may occur when PH follows a glucose load. Importantly, however, this work highlights the paucity of research that has been conducted on this potentially beneficial, low-cost, intervention. Future research should focus on diabetic humans, using a randomised controlled trial design to measure glycaemic control in response to chronic PH. The benefits of PH may follow a dose-response relationship between the temperature and duration of heating [3]. Therefore, future work should determine the appropriate heat exposure to benefit glycaemic control in people with diabetes. If PH is found to be beneficial, then guidelines should then be developed with practical end-user constraints in mind.

## Supporting information

S1 Table PRISMA checklist. (DOC)

## References

[1] Hussain J, Cohen M. Clinical Effects of Regular Dry Sauna Bathing: A Systematic Review. Evidence-Based Complement Altern Med. 2018; 10.1155/2018/1857413 PMC5941775 29849692

[2] Ely BR, Clayton ZS, McCurdy CE, Pfeiffer J, Minson CT. Meta-inflammation and cardiometabolic disease in obesity: Can heat therapy help? Temperature. 2017; 10.1080/23328940.2017.1384089 29687041 PMC5902218

[3] Heinonen I, Laukkanen JA. Effects of heat and cold on health, with special reference to Finnish sauna bathing. Am J Physiol Integr Comp Physiol. American Physiological Society; 2018;314: R629–R638. 10.1152/ajpregu.00115.2017 29351426

[4] Hannuksela ML, Ellahham S. Benefits and risks of sauna bathing. Am J Med. 2001;110: 118–126. 11165553 10.1016/s0002-9343(00)00671-9

[5] Thomas KN. Harnessing heat for health: A clinical application of heat stress. Temperature. 2017; 1–3. 10.1080/23328940.2017.1317379 28944265 PMC5605166

[6] Hooper PL. Hot-Tub Therapy for Type 2 Diabetes Mellitus. N Engl J Med. 1999;341: 924–925. 10.1056/NEJM199909163411216 10498473

[7] Laukkanen T, Khan H, Zaccardi F, JA L. Association between sauna bathing and fatal cardiovascular and all-cause mortality events. JAMA Intern Med. 2015;175: 542–548. 10.1001/jamainternmed.2014.8187 25705824

[8] Zaccardi F, Laukkanen T, Willeit P, Kunutsor SK, Kauhanen J, Laukkanen JA. Sauna Bathing and Incident Hypertension: A Prospective Cohort Study. Am J Hypertens. 2017;30: 1120–1125. 10.1093/ajh/hpx102 28633297

[9] Laukkanen T, Kunutsor S, Kauhanen J, Laukkanen JA. Sauna bathing is inversely associated with dementia and Alzheimer’s disease in middle-aged Finnish men. Age Ageing. 2017;46: 245–249. 10.1093/ageing/afw212 27932366

[10] Kunutsor SK, Khan H, Zaccardi F, Laukkanen T, Willeit P, Laukkanen JA. Sauna bathing reduces the risk of stroke in Finnish men and women. Neurology. 2018; 10.1212/WNL.0000000000005606 29720543

[11] Kunutsor SK, Laukkanen T, Laukkanen JA. Sauna bathing reduces the risk of respiratory diseases: a long-term prospective cohort study. Eur J Epidemiol. 2017;32: 1107–1111. 10.1007/s10654-017-0311-6 28905164

[12] Brunt VE, Howard MJ, Francisco MA, Ely BR, Minson CT. Passive heat therapy improves endothelial function, arterial stiffness, and blood pressure in sedentary humans. J Physiol. 2016;594: 5329–5342. 10.1113/JP272453 27270841 PMC5023696

[13] Brunt VE, Eymann TM, Francisco MA, Howard MJ, Minson CT. Passive heat therapy improves cutaneous microvascular function in sedentary humans via improved nitric oxide-dependent dilation. J Appl Physiol. 2016;121: 716–723. 10.1152/japplphysiol.00424.2016 27418688 PMC6195670

[14] Thomas KN, van Rij AM, Lucas SJE, Cotter JD. Lower-limb hot-water immersion acutely induces beneficial hemodynamic and cardiovascular responses in peripheral arterial disease and healthy, elderly controls. Am J Physiol—Regul Integr Comp Physiol. 2017;312: R28–1R291. 10.1152/ajpregu.00404.2016 28003211

[15] Imamura M, Biro S, Kihara T, Yoshifuku S, Takasaki K, Otsuji Y, et al Repeated thermal therapy improves impaired vascular endothelial function in patients with coronary risk factors. J Am Coll Cardiol. Elsevier Masson SAS; 2001;38: 1083–1088. 10.1016/S0735-1097(01)01467-X 11583886

[16] Kihara T, Biro S, Imamura M, Yoshifuku S, Takasaki K, Ikeda Y, et al Repeated sauna treatment improves vascular endothelial and cardiac function in patients with chronic heart failure. J Am Coll Cardiol. 2002;39: 754–759. 10.1016/S0735-1097(01)01824-1 11869837

[17] Gryka D, Pilch W, Szarek M, Szygula Z, Tota Ł. The effect of sauna bathing on lipid profile in young, physically active, male subjects. Int J Occup Med Environ Health. 2014;27: 608–618. 10.2478/s13382-014-0281-9 25001587

[18] Leppaluoto J, Tuominen M, Vaananen A, Karpakka J, Vuori J. Some cardiovascular and metabolic effects of repeated sauna bathing. Acta Physiol Scand. England; 1986;128: 77–81. 10.1111/j.1748-1716.1986.tb07952.x 3766176

[19] Restaino RM, Walsh LK, Morishima T, Vranish JR, Martinez-Lemus LA, Fadel PJ, et al Endothelial dysfunction following prolonged sitting is mediated by a reduction in shear stress. Am J Physiol Circ Physiol. American Physiological Society; 2016;310: H648–H653. 10.1152/ajpheart.00943.2015 26747508 PMC4796603

[20] Pilch W, Szygula Z, Klimek AT, Palka T, Cison T, Pilch P, et al Changes in the lipid profile of blood serum in women taking sauna baths of various duration. Int J Occup Med Environ Health. Poland; 2010;23: 167–174. 10.2478/v10001-010-0020-9 20682487

[21] Masuda A, Miyata M, Kihara T, Minagoe S, Tei C. Repeated Sauna Therapy Reduces Urinary 8-Epi-Prostaglandin F 2α. Jpn Heart J. 2004;45: 297–303. 10.1536/jhj.45.297 15090706

[22] NCD Risk Factor Collaboration. Worldwide trends in diabetes since 1980: A pooled analysis of 751 population-based studies with 4.4 million participants. Lancet. NCD Risk Factor Collaboration. Open Access article distributed under the terms of CC BY; 2016;387: 1513–1530. 10.1016/S0140-6736(16)00618-8 27061677 PMC5081106

[23] American Diabetes Association. Standards of Medical Care in Diabetes 2017. Diabetes Care. 2017;40.

[24] Zaccardi F, Webb DR, Yates T, Davies MJ. Pathophysiology of type 1 and type 2 diabetes mellitus: a 90-year perspective. Postgrad Med J. 2016;92: 63–69. 10.1136/postgradmedj-2015-133281 26621825

[25] Diabetes Prevention Program Research Group. Reduction in the incidence of type 2 diabetes with lifestyle intervention or metformin. N Engl J Med. 2002;346: 393–403. 10.1056/NEJMoa012512 11832527 PMC1370926

[26] Norris SL, Lau J, Smith SJ, Schmid CH, Engelgau MM. Self-management education for adults with type 2 diabetes: a meta-analysis of the effect on glycemic control. Diabetes Care. 2010;25: 1159–1171. 10.2337/diacare.25.7.1159 12087014

[27] Henstridge DC, Whitham M, Febbraio MA. Chaperoning to the metabolic party: The emerging therapeutic role of heat-shock proteins in obesity and type 2 diabetes. Mol Metab. Germany; 2014;3: 781–793. 10.1016/j.molmet.2014.08.003 25379403 PMC4216407

[28] Stratton IM, Adler AI, Neil HAW, Matthews DR, Manley SE, Cull CA, et al Association of glycaemia with macrovascular and microvascular complications of type 2 diabetes (UKPDS 35): prospective observational study. Br Med J. 2000;321: 405–412. 10.1136/bmj.321.7258.405 10938048 PMC27454

[29] Brenner I, Shek PN, Zamecnik J, Shephard RJ. Stress hormones and the immunological responses to heat and exercise. Int J Sports Med. Germany; 1998;19: 130–143. 10.1055/s-2007-971895 9562223

[30] Jurcovicova J, Vigas M, Palat M, Jezova D, Klimes I. Effect of endogenous GH secretion during hyperthermic bath on glucose metabolism and insulin release in man. Endocrinol Exp. Slovakia; 1980;14: 221–226. 7002532

[31] Laatikainen T, Salminen K, Kohvakka A, Pettersson J. Response of plasma endorphins, prolactin and catecholamines in women to intense heat in a sauna. Eur J Appl Physiol Occup Physiol. 1988;57: 98–102. 10.1007/BF00691246 2830109

[32] Strbák V, Tatár P, Angyal R, Strec V, Aksamitová K, Vigas M, et al Effects of sauna and glucose intake on TSH and thyroid hormone levels in plasma of euthyroid subjects. Metabolism. 1987;36: 426–431. 10.1016/0026-0495(87)90038-2 3106755

[33] Koshinaka K, Kawamoto E, Abe N, Toshinai K, Nakazato M, Kawanaka K. Elevation of muscle temperature stimulates muscle glucose uptake in vivo and in vitro. J Physiol Sci. 2013;63: 409–418. 10.1007/s12576-013-0278-3 23836025 PMC10718043

[34] Baron AD, Steinberg H, Brechtel G, Johnson A. Skeletal muscle blood flow independently modulates insulin-mediated glucose uptake. Am J Physiol. United States; 1994;266: E248–253. 10.1152/ajpendo.1994.266.2.E248 8141283

[35] Hooper PL, Balogh G, Rivas E, Kavanagh K, Vigh L. The importance of the cellular stress response in the pathogenesis and treatment of type 2 diabetes. Cell Stress Chaperones. 2014;19: 447–464. 10.1007/s12192-014-0493-8 24523032 PMC4041942

[36] Padmalayam I. The heat shock response: its role in pathogenesis of type 2 diabetes and its complications, and implications for therapeutic intervention. Discov Med. 2014;18: 29–39. 25091486

[37] Sõti C, Nagy E, Giricz Z, Vígh L, Csermely P, Ferdinandy P. Heat shock proteins as emerging therapeutic targets. Br J Pharmacol. 2005;146: 769–780. 10.1038/sj.bjp.0706396 16170327 PMC1751210

[38] Atalay M, Oksala N, Lappalainen J, Laaksonen DE, Sen CK, Roy S. Heat shock proteins in diabetes and wound healing. Curr Protein Pept Sci. 2009;10: 85–95. 19275675 10.2174/138920309787315202 PMC2743605

[39] Chung J, Nguyen A-KK, Henstridge DC, Holmes AG, Chan MHS, Mesa JL, et al HSP72 protects against obesity-induced insulin resistance. Proc Natl Acad Sci U S A. 2008;105: 1739–1744. 10.1073/pnas.0705799105 18223156 PMC2234214

[40] Strokov IA, Manukhina EB, Bakhtina LY, Malyshev IY, Zoloev GK, Kazikhanova SI, et al The Function of Endogenous Protective Systems in Patients with Insulin-Dependent Diabetes Mellitus and Polyneuropathy: Effect of Antioxidant Therapy. Bull Exp Biol Med. 2000;130: 986–990. 10.1023/A:1002874125993 11177301

[41] Kurucz I, Morva A, Vaag A, Eriksson KF, Huang X, Groop L, et al Decreased expression of heat shock protein 72 in skeletal muscle of patients with type 2 diabetes correlates with insulin resistance. Diabetes. 2002;51: 1102–1109. 10.2337/diabetes.51.4.1102 11916932

[42] Atalay M, Oksala NKJ, Laaksonen DE, Khanna S, Nakao C, Lappalainen J, et al Exercise training modulates heat shock protein response in diabetic rats. J Appl Physiol. American Physiological Society; 2004;97: 605–611. 10.1152/japplphysiol.01183.2003 15075301

[43] Rodrigues-Krause J, Krause M, O’Hagan C, De Vito G, Boreham C, Murphy C, et al Divergence of intracellular and extracellular HSP72 in type 2 diabetes: does fat matter? Cell Stress Chaperones. 2012;17: 293–302. 10.1007/s12192-011-0319-x 22215518 PMC3312959

[44] Bruce CR, Carey AL, Hawley JA, Febbraio MA. Intramuscular Heat Shock Protein 72 and Heme Oxygenase-1 mRNA Are Reduced in Patients With Type 2 Diabetes. Diabetes. 2003;52: 2338–2345. 12941774 10.2337/diabetes.52.9.2338

[45] Krause M, Heck TG, Bittencourt A, Scomazzon SP, Newsholme P, Curi R, et al The chaperone balance hypothesis: The importance of the extracellular to intracellular HSP70 ratio to inflammation-driven type 2 diabetes, the effect of exercise, and the implications for clinical management. Mediators Inflamm. 2015;249205 10.1155/2015/249205 25814786 PMC4357135

[46] Hooper PL, Hooper PL. Inflammation, heat shock proteins, and type 2 diabetes. Cell Stress Chaperones. Dordrecht: Springer Netherlands; 2009;14: 113–115. 10.1007/s12192-008-0073-x 18720028 PMC2727993

[47] Habich C, Sell H, Burkart V. Regulatory Role of Heat Shock Proteins in the Pathogenesis of Type 1 and Type 2 Diabetes. Current Immunology Reviews. 2017 pp. 82–90.

[48] Krause M, Bock PM, Takahashi HK, Homem De Bittencourt PI, Newsholme P. The regulatory roles of NADPH oxidase, intra- and extra-cellular HSP70 in pancreatic islet function, dysfunction and diabetes. Clin Sci. 2015;128: 789–803. 10.1042/CS20140695 25881670

[49] Molina MN, Ferder L, Manucha W. Emerging Role of Nitric Oxide and Heat Shock Proteins in Insulin Resistance. Curr Hypertens Rep. United States; 2016;18: 1 10.1007/s11906-015-0615-4 26694820

[50] Hirosumi J, Tuncman G, Chang L, Görgün CZ, Uysal KT, Maeda K, et al A central role for JNK in obesity and insulin resistance. Nature. Macmillian Magazines Ltd.; 2002;420: 333–336. 10.1038/nature01137 12447443

[51] Yuan M, Konstantopoulos N, Lee J, Hansen L, Li Z-W, Karin M, et al Reversal of Obesity- and Diet-Induced Insulin Resistance with Salicylates or Targeted Disruption of Ikkβ. Science (80-). 2001;293: 1673–1677. 10.1126/science.1061620 11533494

[52] Simar D, Jacques A, Caillaud C. Heat shock proteins induction reduces stress kinases activation, potentially improving insulin signalling in monocytes from obese subjects. Cell Stress Chaperones. 2012;17: 615–621. 10.1007/s12192-012-0336-4 22457223 PMC3535161

[53] Gupte AA, Bomhoff GL, Swerdlow RH, Geiger PC. Heat Treatment Improves Glucose Tolerance and Prevents Skeletal Muscle Insulin Resistance in Rats Fed a High-Fat Diet. Diabetes. 2009;58: 567 LP–578. 19073766 10.2337/db08-1070 PMC2646055

[54] Geiger PC, Gupte AA. Heat Shock Proteins Are Important Mediators of Skeletal Muscle Insulin Sensitivity. Exerc Sport Sci Rev. 2011;39: 34–42. 10.1097/JES.0b013e318201f236 21088604 PMC3670665

[55] Edkins AL, Price JT, Pockley AG, Blatch GL. Heat shock proteins as modulators and therapeutic targets of chronic disease: an integrated perspective. Philosophical transactions of the Royal Society of London. Series B, Biological sciences. 2018 p. 20160521. 10.1098/rstb.2016.0521 29203706 PMC5717521

[56] Krause M, Ludwig MS, Heck TG, Takahashi HK. Heat shock proteins and heat therapy for type 2 diabetes: pros and cons. Curr Opin Clin Nutr Metab Care. 2015;18: 374–380. 10.1097/MCO.0000000000000183 26049635

[57] McCarty MF, Barroso-Aranda J, Contreras F. Regular thermal therapy may promote insulin sensitivity while boosting expression of endothelial nitric oxide synthase—Effects comparable to those of exercise training. Med Hypotheses. Elsevier Ltd; 2009;73: 103–105. 10.1016/j.mehy.2008.12.020 19203842

[58] Miova B, Dimitrovska M, Dinevska-Kjovkarovska S, Esplugues J V, Apostolova N. The Heat Stress Response and Diabetes: More Room for Mitochondrial Implication. Curr Pharm Des. Netherlands; 2016;22: 2619–2639. 10.2174/1381612822666160203114738 26845129

[59] Higgins J, Green S, editors. Cochrane Handbook for Systematic Reviews of Interventions. 5.1.0. The Cochrane Collaboration, 2011; 2011.

[60] Rohatgi A. WebPlotDigitizer 4.1 [Internet]. 2018 [cited 1 May 2018]. https://automeris.io/WebPlotDigitizer

[61] Koivisto VA, Fortney S, Hendler R, Felig P. A rise in ambient temperature augments insulin absorption in diabetic patients. Metabolism. 1981;30: 402–405. 7010077 10.1016/0026-0495(81)90122-0

[62] Tatar P, Vigas M, Jurcovicova J, Jezova D, Strec V, Palat M. Impaired glucose utilization in man during acute exposure to environmental heat. Endocrinol Exp. Slovakia; 1985;19: 277–281. 3910408

[63] Akanji AO, Oputa RA. The effect of ambient temperature on glucose tolerance and its implications for the tropics. Trop Geogr Med. 1991;43: 283–287. 1816663

[64] Rivas E, Newmire DE, Crandall CG, Hooper PL, Ben-Ezra V. An acute bout of whole body passive hyperthermia increases plasma leptin, but does not alter glucose or insulin responses in obese type 2 diabetics and healthy adults. J Therm Biol. Elsevier; 2016;59: 26–33. 10.1016/j.jtherbio.2016.04.010 27264884

[65] Dumke CL, Slivka DR, Cuddy JS, Hailes WS, Rose SM, Ruby BC. The Effect of Environmental Temperature on Glucose and Insulin After an Oral Glucose Tolerance Test in Healthy Young Men. Wilderness Environ Med. Elsevier; 2015;26: 335–342. 10.1016/j.wem.2015.03.002 25937547

[66] Faure C, Charlot K, Henri S, Hardy-Dessources M-D, Hue O, Antoine-Jonville S. Impaired glucose tolerance after brief heat exposure: a randomized crossover study in healthy young men. Clin Sci. 2016;130: 1017–1025. 10.1042/CS20150461 26980346

[67] Frayn KN, Whyte PL, Benson HA, Earl DJ, Smith HA. Changes in forearm blood flow at elevated ambient temperature and their role in the apparent impairment of glucose tolerance. Clin Sci. 1989;76: 323–328. 10.1042/cs0760323 2494016

[68] Linnane DM, Bracken RM, Brooks S, Cox VM, Ball D. Effects of hyperthermia on the metabolic responses to repeated high-intensity exercise. Eur J Appl Physiol. Germany; 2004;93: 159–166. 10.1007/s00421-004-1191-5 15549369

[69] Moses RG, Patterson MJ, Regan JM, Chaunchaiyakul R, Taylor NAS, Jenkins AB. A non-linear effect of ambient temperature on apparent glucose tolerance. Diabetes Res Clin Pract. 1997;36: 35–40. 10.1016/S0168-8227(97)01391-0 9187413

[70] Jezova D, Kvetnansky R, Nazar K, Vigas M. Enhanced neuroendocrine response to insulin tolerance test performed under increased ambient temperature. J Endocrinol Invest. Italy; 1998;21: 412–417. 10.1007/BF03347318 9766253

[71] Akanji AO, Bruce M, Frayn K, Hockaday TDR, Kaddaha GM. Oral glucose tolerance and ambient temperature in non-diabetic subjects. Diabetologia. 1987;30: 431–433. 3678662 10.1007/BF00292547

[72] Koivisto VA. Sauna-induced acceleration in insulin absorption from subcutaneous injection site. Br Med J. England; 1980;280: 1411–1413. 10.1136/bmj.280.6229.1411 PMC1601709 7000239

[73] Koivisto VA. Influence of heat on insulin absorption: Different effects on amorphous and soluble insulins. Acta Diabetol Lat. 1983;20: 174–178. 10.1007/BF02624918 6349204

[74] McGuire EA, Helderman JH, Tobin JD, Andres R, Berman M. Effects of arterial versus venous sampling on analysis of glucose kinetics in man. J Appl Physiol. United States; 1976;41: 565–573. 10.1152/jappl.1976.41.4.565 985402

[75] Blaak EE, Van Baak MA, Kempen KP, Saris WH. Effect of hand heating by a warm air box on O2 consumption of the contralateral arm. J Appl Physiol. United States; 1992;72: 2364–2368. 10.1152/jappl.1992.72.6.2364 1629092

[76] Hawley JA, Lessard SJ. Exercise training-induced improvements in insulin action. Acta Physiol. England; 2008;192: 127–135. 10.1111/j.1748-1716.2007.01783.x 18171435

[77] Beever R. The effects of repeated thermal therapy on quality of life in patients with type II diabetes mellitus. J Altern Complement Med. United States; 2010;16: 677–681. 10.1089/acm.2009.0358 20569036

[78] Heinonen I, Brothers RM, Kemppainen J, Knuuti J, Kalliokoski KK, Crandall CG. Local heating, but not indirect whole body heating, increases human skeletal muscle blood flow. J Appl Physiol. 2011/06/16. American Physiological Society; 2011;111: 818–824. 10.1152/japplphysiol.00269.2011 21680875 PMC3174790

[79] Archer AE, Von Schulze AT, Geiger PC. Exercise, heat shock proteins and insulin resistance. Philos Trans R Soc Lond B Biol Sci. England; 2018;373: 20160529. 10.1098/rstb.2016.0529 29203714 PMC5717529

[80] Kokura S, Adachi S, Manabe E, Mizushima K, Hattori T, Okuda T, et al Whole body hyperthermia improves obesity-induced insulin resistance in diabetic mice. Int J Hyperth. 2007;23: 259–265. 10.1080/02656730601176824 17523018

[81] Bathaie SZ, Jafarnejad A, Hosseinkhani S, Nakhjavani M. The effect of hot-tub therapy on serum Hsp70 level and its benefit on diabetic rats: a preliminary report. Int J Hyperth. 2010;26: 577–585. 10.3109/02656736.2010.485594 20707652

[82] Gupte AA, Bomhoff GL, Touchberry CD, Geiger PC. Acute heat treatment improves insulin-stimulated glucose uptake in aged skeletal muscle. J Appl Physiol. 2011;110: 451–457. 10.1152/japplphysiol.00849.2010 21148343 PMC3043783

[83] Malyshev I, Bayda LA, Trifonov AI, Larionov NP, Kubrina LD, Mikoyan VD, et al Cross-talk between nitric oxide and HSP70 in the antihypotensive effect of adaptation to heat. Physiol Res. Czech Republic; 2000;49: 99–105. 10805410

[84] Iguchi M, Littmann AE, Chang S-H, Wester LA, Knipper JS, Shields RK. Heat Stress and Cardiovascular, Hormonal, and Heat Shock Proteins in Humans. J Athl Train. 2012;47: 184–190. 22488284 10.4085/1062-6050-47.2.184 PMC3418130

[85] Whitham M, Laing SJ, Jackson A, Maassen N, Walsh NP. Effect of exercise with and without a thermal clamp on the plasma heat shock protein 72 response. J Appl Physiol. American Physiological Society; 2007;103: 1251–1256. 10.1152/japplphysiol.00484.2007 17673560

[86] Gibson OR, Dennis A, Parfitt T, Taylor L, Watt PW, Maxwell NS. Extracellular Hsp72 concentration relates to a minimum endogenous criteria during acute exercise-heat exposure. Cell Stress Chaperones. Netherlands; 2014;19: 389–400. 10.1007/s12192-013-0468-1 24085588 PMC3982022

[87] Kenny GP, Sigal RJ, McGinn R. Body temperature regulation in diabetes. Temperature. 2016;3: 119–145. 10.1080/23328940.2015.1131506 27227101 PMC4861190

[88] Al-Qaissi A, Papageorgiou M, Javed Z, Heise T, Rigby AS, Garrett AT, et al Environmental effects of ambient temperature and relative humidity on insulin pharmacodynamics in adults with type 1 diabetes mellitus. Diabetes Obes Metab. 2018;Epub. 10.1111/dom.13555 30311402

[89] Krämer HH, Rolke R, Bickel A, Birklein F. Thermal Thresholds Predict Painfulness of Diabetic Neuropathies. Diabetes Care. 2004;27: 2386 LP–2391. 10.2337/diacare.27.10.2386 15451905

